# On the effect of chess training on scholastic achievement

**DOI:** 10.3389/fpsyg.2014.00762

**Published:** 2014-08-08

**Authors:** William M. Bart

**Affiliations:** Department of Educational Psychology, University of MinnesotaMN, USA

**Keywords:** chess, cognition, education, scholastic achievement, expertise

What are the effects of chess training—especially on scholastic achievement among school-aged students? Can chess instruction facilitate the acquisition of scholastic competency? The current state of the research literature is that chess training tends not to provide educational benefits. This article provides a critical review of research on the effects of chess training on the scholastic achievement levels of school-aged students.

## Educational benefits of chess

Various studies and conference presentations (e.g., Christiaen and Verholfstadt, 1978; Liptrap, 1998; Bart and Atherton, 2004) provided results in support of the educational benefits of chess instruction in the schools. Gobet and Campitelli (2006) reviewed that research and reached the following conclusions: (a) the educational effects of optional scholastic chess training remain undetermined; (b) compulsory scholastic chess instruction may engender motivational problems among students; and (c) chess instruction may be beneficial among novices, but is less important among intermediate and advanced players for whom the amount of practice and the acquisition of knowledge are of paramount importance. Gobet et al. (2004) contended that such conclusions are in line with the view of de Groot (1977, 1978) that educational benefits of chess instruction are likely “low-level gains” such as improvements in attention and concentration and interest in learning, rather than “high-level gains” such as improvements in intelligence, scholastic achievement, and creativity.

Additional research is supportive of de Groot's view. Bilalić et al. (2007) determined that intelligence explained a smaller amount of the variance in chess skill among competent young chess players than the amount of practice time. Waters et al. (2002) also found little support for a relationship between intelligence and chess skill.

Contrary to the results of Gobet and Campitelli (2006) that chess instruction provides very modest if any educational benefits is research that attests to the benefits of chess training. For example, Smith and Cage (2000) reported the effects of 120 h of chess instruction on the mathematics achievement among rural, African-American secondary school students in northern Louisiana. They determined that the treatment group composed of 11 females and 10 males scored significantly higher in mathematics achievement and non-verbal cognitive ability than the control group composed of 10 females and 10 males after controlling for differences among pretest scores.

In a more recent study Aciego et al. (2012) used a quasi-experimental study to examine the cognitive effects of chess training. The experimental group consisted of 170 students, 6–16 years of age, who received extracurricular chess instruction. The comparison group consisted of 40 students in a similar age range. Those students received extracurricular sports (soccer or basketball) activities. The Wechsler Intelligence Scale for Children (WISC-R) and a record completed by the tutor-teacher to measure problem solving were dependent variables.

After adjusting for pretest scores, the chess group registered significantly higher posttest scores than the sports group for five of nine WISC-R subtests—i.e., the Similarities, Digit Span, Block Design, Object Assembly, and Mazes subtests. The chess group also registered significantly higher posttest scores in problem solving capacity than the sports group. The authors concluded that chess is a “valuable educational tool” (p. 558).

In another recent study Kazemi et al. (2012) examined the cognitive effects of chess play. They employed an experimental group composed of 86 randomly selected school-aged students, who received chess instruction for six months, and a control group of 94 randomly selected school-aged students. All participants were male and from 5th, 8th, and 9th grades from schools in Shanandaj in western Iran. All participants were administered a measure of metacognitive ability and a grade-appropriate mathematics exam prior to and after the intervention.

The chess group participants registered significantly higher posttest metacognitive ability scores and higher posttest mathematics test scores than the non-chess group participants. A major conclusion of the study is that chess instruction improves significantly the mathematical abilities and the metacognitive capacities of school-aged students.

In a third study, Trinchero (2013) examined the effects of chess instruction on the mathematical ability of primary school students. His study involved 568 primary school children in Italy placed in four groups: (1) experimental, (2) control, (3) experimental without a pretest, and (4) control without a pretest. The experimental group received chess training in addition to ordinary class lessons. The control group only received ordinary class lessons. One prominent result was that the experimental group that received chess training registered a modest but statistically significant increase in scores on mathematics test items that required problem-solving skills on complex tasks. That effect was greater among students who had more hours of chess instruction.

These last four studies lend support to the view that chess training has positive cognitive effects on regular school-aged students. In addition, there are some studies that address the issue of cognitive effects of chess training on school-aged students with disabilities.

Scholz et al. (2008) investigated the effects of chess training on mathematics learning among students with learning disabilities based on intelligence scores in the 70–85 IQ range. School classes from four elementary schools in Germany were randomly assigned to two groups: (a) an experimental group that received chess instruction of one hour per week for one entire school year; and (b) a comparison group that received supplementary mathematics instruction of one hour per week. The two groups did significantly differ in their calculation abilities for simple addition tasks and counting. The authors concluded “chess could be a valuable learning aid for children with learning disabilities” (p. 138).

In a second study Barrett and Fish (2011) investigated the cognitive effects of a 30-week chess-training program within mathematics classes for students in special education in a middle school in southwestern United States. All participants qualified for special education services and were in either 6th, 7th, or 8th grades. A sample of 31 participants were randomly placed into two groups: (a) an experimental group composed of 15 students who received the chess instruction along with a sizable portion of the regular instruction in resource mathematics specially designed for students in special education; and (b) a comparison group composed of 16 students who received all of the regular mathematics instruction in resource mathematics rather than any chess instruction. The dependent variables for this study were scores on the mathematics Texas Assessment of Knowledge and Skills (TAKS) that is a standardized test of mathematics competencies for pre-collegiate students in Texas, and end-of-year course grades in resource mathematics. All participants completed a version of the mathematics TAKS that was modified for special education students.

This study had some interesting results. First, there was a significant relationship between chess instruction and end-of-year grades. Second, there was a statistically significant relationship between chess instruction and mathematics TAKS test scores.

This study provided support that chess instruction facilitates transfer of cognitive skills from chess to mathematics for students in special education.

These latter two studies using special education students indicate that chess instruction has the potential to promote mathematics achievement among students in special education.

In a third study Hong and Bart (2007) examined the cognitive effects of chess instruction on students at risk for academic failure in Korea. The total sample of participants was 38 students from three elementary schools randomly placed into two groups: (a) an experimental group that received 90-min chess lessons weekly for 3 months; and (b) a comparison group that received regular school activities after class.

All participants were tested prior to and after the intervention with the following several instruments: (a) the Test of Nonverbal Intelligence—Third Edition (TONI-3) to measure cognitive ability in a language-free manner; and (b) a Chess Skill Rating method using chess software to assess level of chess competency.

TONI-3 posttest scores and chess skill ratings were significantly correlated after controlling for TONI-3 pretest scores. This statistical finding suggests that chess instruction that produces higher chess skill ratings may lead to gains in levels of non-verbal intelligence among students at risk for academic failure.

These studies indicating positive effects of chess training among students with disabilities support the view of Storey (2000) who extolled the use of chess training as a means to promote higher-order thinking skills among disabled students. Storey (2000, p. 47) recommended that teachers consider “chess as an instructional strategy for reinforcing skills such as concentration, problem identification, problem solving, planning strategies, creativity, and lucid thinking.”

But why would chess training lead to improvements in scholastic achievement? To play chess well, one must attend to and comprehend chess positions and induce patterns among the pieces, an indication of fluid intelligence and concentration capacity. The chess positions can be very complex with up to 32 pieces from six piece types arrayed on a 64 square board.

One must then formulate and evaluate possible moves, an indication of executive functioning and critical thinking. For example, middle game positions often permit 30 different legal moves at every turn. The chess player must ideally evaluate positions resulting from such moves selecting the move that produces the position most advantageous to the player. The chess player must evaluate chess moves and their resulting positions without actually moving any pieces. There are thus substantial demands on visual working memory.

In chess, one must engage in this sequence of (1) position comprehension, (2) pattern induction, and (3) move formulation and evaluation relatively quickly. This coordinated set of cognitive skills required in competent chess play likely transfers to the learning of mathematics and related fields that also often require comprehension, induction, analysis, and evaluation of complex phenomena. This constitutes a theoretical framework why chess training likely has cognitive benefits. This cognitive explanation for the benefits of chess training is compatible with comparable explanations provided by Storey (2000) and Trinchero (2013).

## Chess training, expertise, and the issue of research rigor

The research reported thus far provides evidence that chess training has salutary cognitive and educational effects among school-aged students. However, the argument of Gobet and Campitelli (2006) needs to be considered before we can be confident that chess training is a valid means to improve scholastic achievement levels. To Gobet and Campitelli (2006), rigorous experimental research is needed to determine the extent to which chess training has strong cognitive and educational effects.

However, such rigorous experimental inquiry involving, for example, random placement of participants into experimental and control groups is costly and difficult to implement in a school setting. What is needed is an increase in the quality and quantity of empirical studies to determine the extent to which the acquisition of chess expertise facilitates the acquisition of scholastic expertise among students.

### Conflict of interest statement

The author declares that the research was conducted in the absence of any commercial or financial relationships that could be construed as a potential conflict of interest.
